# Exploring the Factors That Influence Stakeholder Participation in Decision-Making for the Moat System Restoration Project in Tianchang City, China

**DOI:** 10.1155/2023/9969589

**Published:** 2023-11-11

**Authors:** Shihua Yan, Roziya Binti Ibrahim

**Affiliations:** Department of Landscape Architecture, Faculty of Design & Architecture, Universiti Putra Malaysia, Serdang 43400, Selangor, Malaysia

## Abstract

The driving factors have a critical effect on shaping stakeholder behavior toward participating in decision-making for river restoration initiatives. The participation of stakeholders is a vital determinant for increasing public confidence in the government and enhancing the acceptance of government decisions. Conversely, insufficient stakeholder participation in decision-making may lead to resistance to decisions on river restoration projects. Thus, the primary purpose of this investigation is to shed light on the complex interactions between the various drivers that underpin stakeholder participation in the context of the Moat System Restoration Project (MSRP). The extended Theory of Planned Behavior (TPB) describes the relationships between seven drivers that have positively influenced stakeholder participation behaviors: stakeholder attitude, priority, risk perception, trust in government decisions, motivation, intention, and knowledge. The empirical underpinning of this research was obtained through a questionnaire survey conducted in Tianchang, China, encompassing a sample size of 473. The empirical findings discern that stakeholder attitudes vis-à-vis the MSRP favorably influence stakeholder participation behaviors. Additionally, stakeholder motivation and intention have been discerned as catalysts for heightened stakeholder participation behavior. These findings promise to furnish invaluable insights, benefit forthcoming river restoration initiatives, and equip decision-makers with a profound understanding of strategies to enhance stakeholder participation.

## 1. Introduction

The persistently high frequency of floods has brought unstoppable challenges to human society [1]. Evidently, in 2021, China's Henan Province suffered massive flooding, resulting in 398 deaths and a direct economic loss of 120.6 billion yuan with substantial damage. Furthermore, recent spates of recurrent flooding have left a trail of devastation, encompassing property damage, displacement [2], and the insidious emergence of diseases and mental health issues [3]. However, in the face of assiduous efforts, scarcely any nation or region remains immune to the ravages wrought by incessant deluges [4]. In 2050, 1.6 billion individuals will be exposed to the perils of flooding, according to the United Nations Conference on Environment and Development [5]. In the context of this severe test, the term “river restoration” has become a magic bullet for effective flood management [6]. The term is commonly used to describe the behavior of river courses, adjacent riparian zones, and floodplains [7]. The formulation of river restoration objectives necessitates the solicitation of stakeholder voices and the negotiation of restoration objectives that secure the consensus of a preponderance of stakeholders. The International Principles and Standards for Ecological Restoration Practice [8] underscore the importance of heeding stakeholder aspirations and encouraging their direct engagement. Integrating stakeholder input provides a channel for decision-makers to identify restoration goals that are consistent with stakeholder expectations while enhancing stakeholder understanding of the risks and benefits associated with river restoration initiatives. Several literature studies introduce cases of public participation in river restoration projects, such as the case studies of “Room for the River Project,” “Ythan River Restoration Project,” and “Hudson River Restoration Project” [9–11].

In the Chinese context, Moat Systems assume a distinctive role by preserving the cultural heritage and augmenting ecological resilience [12]. Concretely, a moat represents an artificial waterway encircling an entire city, functioning as an extension of the city's fortifications [13]. Historically, moats in ancient China primarily served the dual purpose of flood control and urban defense. Nonetheless, as posited by Darling and Abontaen-Eghafona [14], residents may have perceived Moat Systems as superfluous infrastructure characterized by a single functional dimension. Numerous urban Moat Systems have borne witness to a litany of issues, including recurrent flooding, pollution, and deteriorating water quality, thereby giving rise to a host of social predicaments. In light of this, the present investigation endeavors to dissect the intricate interplay among the determinants propelling stakeholder participation in the decision-making processes underpinning the Moat System Restoration Project. In the extant literature, “stakeholders” have four distinct typologies, including those directly impacted by decisions and those vested with the capacity to sway ultimate determination [15]. Stakeholders also encompass individuals or groups capable of exerting positive or negative influence on decision implementation, alongside those possessing a vested interest or stake in the issue at hand [16]. This study aligns with Freeman's [17] formulation of stakeholders as those who live or work close to the Moat System, including local authorities, academics, and relevant organizations. Their lives will be affected by the MSRP, and they simultaneously have the right and the capability to participate in the decision-making.

Previous academic research has examined the connection between stakeholder participation and river restoration through different lenses, such as investigating how attitude [18], risk perception [19], priority [20], trust in government [21], and knowledge [22] relate to stakeholder participation behavior. Likewise, Marsh et al. [23] summarize the benefits of voluntary participation in restoration projects, such as how stakeholders should be involved in decision-making [24] and how stakeholders can be better involved in river restoration projects [25]. However, the research found that very few studies have directly investigated the relationship between the driving factors behind stakeholder participation in decision-making for river restoration projects. To bridge this gap, the present research employed an empirical investigation method that combined the drivers summarized in previous research, including stakeholder attitudes, priorities, risk perceptions, trust in government, motivations, intentions, and knowledge, aiming to examine associations between these drivers. The insights garnered hold the potential to furnish decision-makers with a comprehensive understanding of the prevailing challenges within the Moat System, thereby equipping local authorities with the requisite information to foster judicious decision-making.

## 2. Literature Review

### 2.1. Stakeholder Participation in Decision-Making

The efficacy of river restoration projects hinges, to a certain extent, on the voluntary participation and commitment of stakeholders [26]. Participation not only provides all relevant stakeholders with the opportunity to become familiar with the river restoration project but also has the important significance of helping them realize the value of their participation. Stakeholder empowerment also contributes to elevating the transparency of the decision-making process and upholding the principles of fairness and legitimacy [27]. However, in China, stakeholders are frequently precluded from participating in and influencing decision-making processes [28]. Conniff [29] reports that roughly 75% of executed river restoration projects fall short of their objectives, largely attributable to inadequate stakeholder participation. A notable constraint lies in the dearth of requisite knowledge among individuals, limiting their participation [30]. Skepticism towards government and a limited knowledge base pose formidable impediments to stakeholder participation.

To fulfill the research objectives and to be easily understood, in this study, “attitude” can be construed as stakeholders' response to the Moat System Restoration Project (MSRP), while “stakeholder participatory behavior” represents the implementation of participatory behavior by stakeholders. “Motivation” characterizes what drives stakeholders to voluntarily participate in decision-making, while “intention” indicates the desired outcome that stakeholders hope to achieve through their participation. “Risk perception” denotes stakeholders' assessment of the risk level associated with the Moat System, while “priority” alludes to their preferred primary restoration target within MSRP. “Stakeholder knowledge” reflects their familiarity with knowledge pertinent to participation in decision-making.

Motivation is a prerequisite for people to consistently participate [31]. Without motivation, people are less likely to participate in decision-making [32]. Motivation consists of internal and external factors [33]. Previous studies have shown that the intrinsic motivation behind voluntary participation often comes from individual social responsibility [34], moral obligation [35], and self-confidence [36], while the extrinsic motivation is often driven by obtaining rewards and avoiding negative consequences [37]. Intrinsic motivation represents the behavior of participation as inherently interesting and satisfying [38]. Several scholars have also examined stakeholders' intentions to participate in decision-making and found that most were to establish valuable professional contacts [39], satisfy curiosity [40], acquire knowledge [22], and make new friends [41]. Bouazzaoui and Daniels's [42] survey respondents stated, “*I participate because I want my voice to be heard*.”

Lechowska [43] highlighted the importance of understanding the flood risk perceptions of individuals in flood-prone areas, as they often exhibit poor risk awareness and underestimate potential hazards, thereby weakening their willingness and motivation to participate [44]. Risk perception was an important factor that caused differences in behavioral intentions toward climate change [45]. Investigating the factors that influence risk perceptions helps decision-makers understand people's reactions to risk events and address the problem of people underestimating flood risk [46]. Intriguingly, Xu [47] discerned that heightened governmental trust correlated with diminished risk perception. When people judge risks in the absence of relevant knowledge, they will rely on trust in government decisions. If the level of perceived risk continues to rise, governments will face a crisis of trust. On the other hand, Ker Rault et al. [48] argue that the nonavailability of relevant knowledge was one of the main barriers to participation. Over half of the respondents expressed a lack of knowledge regarding the participatory process [49]. In this context, Cundill and Rodela [50] introduced a social learning paradigm aimed at augmenting stakeholders' problem-solving competencies, bridging the chasm between collaborative multistakeholder learning and the generation of novel knowledge.

At present, priority approaches have been widely implemented in river restoration projects [51]. The concept of “priority” in river restoration means that stakeholders rank river restoration objects in order of importance [52]. Even resolving the discrepancy between the decision-makers' ideas and social preferences in the decision-making process is not an easy task. In this study, “stakeholder priorities” can be considered as stakeholder perceptions of the first restoration target in the MSRP. Friedman et al. [53] suggested that priority restoration goals could be identified based on the attributes of stakeholders and their attitudes or behaviors toward their MSRP. In recent decades, several scholars have analyzed how to determine the priority targets for river restoration; for example, Uribe et al. [54] surveyed the general public, academia, NGOs, and government officials to identify their priorities. Gallego-Ayala and Juízo [55] applied an analytic hierarchy process and successfully integrated stakeholder priorities into river restoration projects. In summary, it is recommended that stakeholders' attitudes, knowledge, risk perceptions, trust in government decisions, and priorities be incorporated into the MSRP decision-making process. Ideally, decision-makers, stakeholders, and experts actively communicate and negotiate to reach a consensus on the final MSRP decision or alternative.

### 2.2. The Theory of Planned Behavior (TPB)

The Theory of Planned Behavior (TPB), devised by Ajzen [56], pivots on the concept of individual intention to execute a specific behavior. TPB posits that behavioral intention is the primary antecedent of an individual's actions, with the strength of intention directly influencing the likelihood of behavior occurrence. Attitudes toward behavior, subjective norms, and perceived behavioral control constitute a triad of factors influencing individual behavioral intentions. “Attitude” signifies an individual's positive or negative evaluation of a certain object, and “subjective norm” pertains to the individual's perception of societal pressures, either facilitating or impeding the enactment of the behavior. Perceived behavioral control encompasses an individual's assessment of the ease or difficulty associated with the targeted behavior.

With the application of TPB, the study attempted to explore the associations observed with diverse drivers that promote stakeholder participation in decision-making for river restoration projects. However, it must be acknowledged that some scholars have questioned the explanatory power of the TPB regarding people's participation behavior. Thus, this research applies the extended TPB framework by incorporating additional variables such as stakeholder knowledge, trust in government, risk perception, and priority as a means of elucidating the relationship between these factors to help motivate stakeholders to participate in river restoration projects.

### 2.3. Conceptual Framework Development

#### 2.3.1. Knowledge

Individual knowledge, attitudes, and participation behaviors exhibit intricate interconnections [57]. Knowledge demonstrates a positive correlation with attitudes, and empirical evidence by Zelezny [58] corroborates the influence of knowledge on behavior. Individuals endowed with knowledge of decision-making participation exhibit heightened likelihood of engaging in such behaviors [59]. Knowledge also bears significance in shaping risk perception [60], which in turn forecasts stakeholders' behavioral motivations and intentions [61] and informs stakeholder priorities [62]. Hence, the following hypotheses are posited:

**Hypothesis 1 .**

Stakeholder knowledge has a positive impact on participation behavior.

**Hypothesis 2 .**

Stakeholder knowledge has a positive impact on perceived risk.

**Hypothesis 3 .**

Stakeholder knowledge has a positive impact on attitude.

**Hypothesis 4 .**

Stakeholder knowledge has a positive impact on priorities.

#### 2.3.2. Trust in Government

Trust is a state of mind that includes the intention to accept vulnerability based upon positive expectations of the intentions or behavior of another [63] and plays a pivotal role in determining whether individuals permit external influences on their behavior [64]. Moreover, trust has been identified as a crucial predictor of perceived risk [65], with low trust often correlating with heightened risk perceptions. The relationship between trust and attitude is well-established [66]. Consequently, the following hypotheses are advanced:

**Hypothesis 5 .**

Stakeholder trust in government has a positive impact on participation behavior.

**Hypothesis 6 .**

Stakeholder trust in government has a positive impact on attitude.

**Hypothesis 7 .**

Stakeholder trust in government has a positive impact on perceived risk.

#### 2.3.3. Risk Perception

Risk perception, denoting individuals' subjective assessment of potential risks [46], demonstrates a nexus with attitudes [67]. Risk perception informs individuals' attitudes toward hazards, consequently influencing behavioral intentions [68] and priorities [69]. Therefore, the following hypotheses are posited:

**Hypothesis 8 .**

Stakeholder risk perception has a positive impact on participation behavior.

**Hypothesis 9 .**

Stakeholder risk perception has a positive impact on attitude.

**Hypothesis 10 .**

Stakeholder risk perception has a positive impact on intention.

**Hypothesis 11 .**

Stakeholder risk perception has a positive impact on priority.

#### 2.3.4. Priority

Collective decision-making based on the individual's preferences and priorities is considered relevant to democratic institutions [70]. Pratkanis et al. [71] described the association with stakeholders' priorities and behaviors from the TPB perspective. Individuals' preferences were positively interrelated with participation frequency [72]. Moreover, Jennings et al. [73] state that priorities and attitudes are positively correlated. Understanding one's motivations helps him or her set priorities [74]. Therefore, this research proposes the following hypotheses:

**Hypothesis 12 .**

Stakeholder priority has a positive impact on participation behavior.

**Hypothesis 13 .**

Stakeholder priority has a positive impact on attitude.

**Hypothesis 14 .**

Stakeholder priority has a positive impact on motivation.

#### 2.3.5. Motivation and Intention

The TPB proposes that intentions are an important factor influencing individual motivation and behavior [56]. In TPB, intentions are assumed to capture the motivational factors that influence a behavior. Both intrinsic and extrinsic motivations were significantly associated with intention [75]. Intentions may also affect people's attitudes. Meanwhile, Rafi'ah et al.'s [76] empirical research found a correlation between people's intentions and behavior. When stakeholders are motivated, they are more likely to participate in decision-making. With reference to the above, the following assumptions are made:

**Hypothesis 15 .**

Stakeholder motivation has a positive impact on participation behavior.

**Hypothesis 16 .**

Stakeholder motivation has a positive impact on intention.

**Hypothesis 17 .**

Stakeholder intention has a positive impact on participation behavior.

**Hypothesis 18 .**

Stakeholder intention has a positive impact on attitude.

#### 2.3.6. Attitude

The term “attitude” refers to people's evaluation of an object in a like-or-dislike manner [77]. Attitudes cause behavior [78]. If somebody's attitude changes, their intentions are likely different, and subsequent behavior will be affected [79]. Ajzen and Fishbein [80] believe that attitudes play an important role in motivating stakeholders to perform participation behaviors. There was a positive correlation between active attitudes and participation [81]. Thus, we posit the following hypotheses:

**Hypothesis 19 .**

Stakeholder attitude has a positive impact on participation behavior regarding the river restoration project.

**Figure 1:**
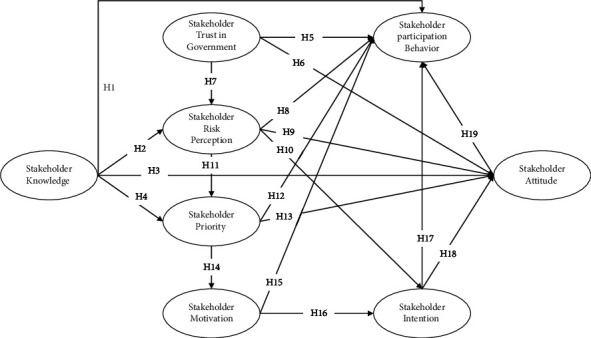
Conceptual framework.

In summary, Figure 1 illustrates the hypothesized relationships among these variables.

## 3. Method

### 3.1. Study Area

**Figure 2:**
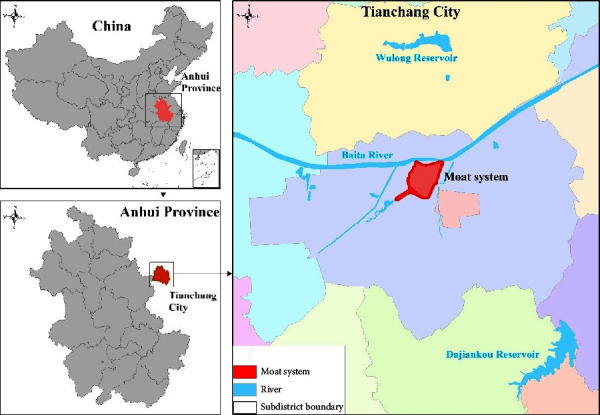
Location of the Moat System in Tianchang city.

Tianchang city (TC) is located in Anhui Province, China. It is a county-level city of approximately 620,000 inhabitants with a subtropical monsoon climate. The Moat System flows around the entire TC (see Figure 2). The Moat System plays a valuable role as the green infrastructure of TC in the provision of ecosystem services and human well-being. However, frequent concentrated rainfall and urban development have caused considerable damage to TC in recent decades, especially to residents who live or work near the Moat System. The Anhui Provincial Government Flood Investigation and Assessment Team reported that, in 2021, the flooding affected 10,465,300 people and caused 14 deaths.

Although the TC government has launched several response initiatives, the countermeasures have not always been well received, leading to a consistent decline in people's trust in government decisions. In this case, to address this problem, the Moat System Management Team, as a representative of the TC government, launched the “MSRP” in 2016. The project seeks to motivate people to participate in MSRP decision-making to capture stakeholder ideas and feedback so that the final MSRP decision is more acceptable. In 2017, the members of the Moat System Management Team conducted several simple conversations with the resident groups living near the Moat System. The Moat System Manager simultaneously established a dedicated mailbox to understand stakeholders' thoughts, complaints, and expectations on MSRP, thereby motivating people's desire to participate in MSRP decision-making.

### 3.2. Measurement Instrument

The measurement items for the hypothesized constructs in this research were developed based on the existing body of available literature. The stakeholder knowledge measurement item was taken from Buchecker et al. [82] and Buchecker [83], while stakeholder trust in government was derived from Mah et al.'s [84] scale with minor modifications to suit the unique context of the current study. Stakeholder priorities were adapted from the five-item scale from Beechie et al. [85] and Patrik Berander [86]. In addition, to measure stakeholder risk perception, the researcher referenced the scale developed by Su et al. [87] and Whitmarsh [88] to reflect how stakeholders react to risk. For example, “positive attitudes and resilience towards disaster recovery” and “climate change is an important factor that scares me.” Moreover, the measurement of stakeholder motivation for participation drew upon a 5-item scale from Mah et al. [84]; Corbett [89]; and van Riper [36]. The dimension of stakeholder participation intentions was adapted from Woosnam et al. [90] and Venkataramanan et al. [91]. Items adapted from Wang et al. [92] and Sarvilinna [93] were used to measure stakeholder participation behavior.

It is noteworthy that all measurement scales employed in this study are unidimensional and presented in a five-point Likert format, where the numerical values “1” and “5” correspond to strongly disagree and “strongly agree,” respectively. In addition to addressing fundamental sociodemographic inquiries, the questionnaire incorporated several open-ended questions at its conclusion, affording participants the opportunity to articulate their perspectives with greater depth and granularity.

### 3.3. Data Collection

This investigation utilized specific sampling techniques for diverse population groups. The people who live or work around the Moat System, government departments, the Moat System Management Team, experts and relevant organizations, and all those interested in the MSRP were the principal participants of this research. Before undertaking the present study, this research had obtained permission from the Tianchang Municipal government to ensure the data collection could proceed successfully. During the data collection process, the researcher provided each participant with a consent form to read and understand their rights before participating in the study, such as their right to withdraw at any time and at any stage.

Researchers utilized specialized sampling techniques for different groups to profile their unique characteristics. (1) People who live near the Moat System use convenience sampling. Convenience sampling is a type of nonprobability sampling approach. The most significant advantage is the simplicity, quickness, and relatively inexpensive cost of recruiting participants [94]. This method often collects samples from individuals who are geographically accessible, recruitable, and/or willing to participate in the study. (2) Government Agencies and Moat System Management Teams use purposeful sampling. This technique helps to gain insights from key stakeholders responsible for decision-making and implementation. (3) Experts use a combination of purposive and snowball sampling, which is preferable. Purposive sampling involves selecting participants based on expert domain and relevance to this study [95]. The technique can help the researcher identify different experts whose perspectives are cross-disciplinary and intellectual boundaries. (4) Related organizations using the representative sample method help the researcher select organizations that play a critical role in MSRP or have specific knowledge within the field, ensuring a balanced expression of perspectives [96].

The data were collected from October to December 2022. Most questionnaires were completed by scanning QR codes on WeChat. On-site data collection was utilized for those unfamiliar with online surveys. The researchers received a total of 510 questionnaires, including 368 online and 142 offline questionnaires. During the data screening process, 37 invalid questionnaires were eliminated. Therefore, 473 valid questionnaires were obtained for analysis, and the response rate was 92.55%.

### 3.4. Data Analysis

All data were entered in SPSS 25.0 to undertake a descriptive statistical analysis of the amassed data. Structural equation modeling (SEM) was adopted to test the proposed research hypotheses, facilitated by the AMOS 26.0 software. SEM is a commonly used technique to test models with observed and latent variables [97]. In the conceptual framework, behavior is classified as the dependent variable, while attitude and other factors are classified as the independent variables. A two-step procedure is adopted to test the research hypotheses in this research [98]. Specifically, confirmatory factor analysis is used to estimate the reliability and validity of the constructs, and path analysis is used to test hypothesized causal structures between variables. We applied several fit indices to assess model fit, such as the ratio of Chi-square to degrees of freedom, the Goodness-of-Fit Index (GFI), the Comparative Fit Index (CFI), and the Normative Fit Index (NFI).

## 4. Results

### 4.1. Characteristic Respondent

Table 1 provides an insightful demographic profile of the study participants, thereby affording a comprehensive snapshot of the composition of the 473 individuals who constituted the research cohort. It is discerned that the gender distribution was relatively balanced, with males and females contributing 50.7% and 49.3%, respectively, to the respondent pool. A minority of participants belong to the 46- to 55-year-old cohort (18.2%), whereas 17.1% fall within the age range of 36 to 45 years.

Of particular note is the participants' educational attainment. Conspicuously, 37.6% of respondents have attained the bachelor's degree level, while appreciable 18% have acquired a master's degree, and select 4.2% have a doctorate. In contrast, the aggregate encompassing primary and secondary educational attainments collectively accounted for 40.2% of the participants. In the realm of employment status, a diverse spectrum emerges. Civil servants constitute substantial 39.7% of the participant cohort. By juxtaposition, workers account for 18.4%, scholars for a modest 1.7%, and the self-employed stake claims an 18% share. The remaining demographic cohort comprises 22.2%.

It is noteworthy that 22% of participants have lived or worked near the moat system for over 20 years. Participants who had inhabited this locale for durations ranging from 8 to 13 years and 14 to 19 years accounted for 18.6% and 18.4%, respectively. Meanwhile, 21.1% had lived there between 2 and 7 years. 19.9% of respondents had inhabited the vicinity surrounding the Moat System for less than a year. Turning to familiarity with knowledge about participation in decision-making, 20.5% of stakeholders have a very high level of familiarity (extreme familiarity), while 20.3% can be categorized as “very familiar.” 19.5% were in the “moderately familiar” range, while 22.2% were “slightly familiar.” Only a small minority (17.5%) indicated that they were “not at all familiar” with the complexity of participating in decision-making.

The research also gauged participants' perspectives about priority goals for the MSRP. It was observed that as many as 23.9% of respondents strongly approved of “flood management” as a top MSRP priority, whereas 22.2% espoused diametrically opposing views, strongly disapproving of this thought. An additional 16.5% of the participants assumed a neutral stance. However, attitudinal variance persists, with 19.2% disapproving and 18.2% approving of this priority.

### 4.2. Measurement Model and CFA

In this study, Cronbach's alpha was used to examine the internal consistency between different items. Composite reliability and average variance extracted (AVE) values represent construct validity. As shown in Table 2, Cronbach's alpha coefficients for all constructs ranged from 0.905 to 0.811, exceeding the value of 0.8, which is considered good. Factor loadings for all items were above the recommended benchmark of 0.70 [99]. Composite reliability scores ranged from 0.902 to 0.841, all above the acceptable value of 0.70 [100]. Furthermore, all AVE scores were above 0.6, indicating adequate convergent validity [101], and discriminant validity was confirmed as the AVE value for each construct was found to be greater than the square of the correlation between the corresponding constructs (Table 3). For instance, the AVE square root value of stakeholder knowledge was 0.804, which was greater than the maximum value of the absolute value of the correlation coefficient between factors of 0.466. The AVE square root value of stakeholder trust in government was 0.778, which was greater than the maximum value of the absolute value of the correlation coefficient between factors of 0.506, indicating that it had good discrimination validity. Thus, given these results, it can be concluded that the measurement model has sufficient reliability, convergent validity, and discriminant validity.

The fit of the model was tested by Chi-square statistics, the Comparative Fit Index (CFI), the Tucker–Lewis Index (TLI), the approximate root mean square error (RMSEA), and the standardized root mean square residual (SRMR).  Table 4 shows that the proposed models have an acceptable overall fit, that is, the measurement model (Chi-square = 762.402; df = 674; Chi-square/df = 1.131; SRMR = 0.031; RMSEA = 0.017; TLI = 0.991; CFI = 0.992) and the structural model (Chi-square = 791.242; df = 682; Chi-square/df = 1.160; SRMR = 0.054; RMSEA = 0.018; TLI = 0.989; CFI = 0.990).

### 4.3. Structural Model and Hypothesis Testing

The measurement model was turned into a structural model by adding hypothesized paths between the constructs. As shown in Table 5, results indicated that the standardized path coefficient from stakeholder knowledge to stakeholder behavior was 0.248 (*p* < 0.001) (H1 supported), stakeholder risk perception was 0.386 (*p* < 0.001) (H2 supported), stakeholder attitudes were 0.140 (*p* < 0.01) (H3 supported), and stakeholder priorities were 0.292 (*p* < 0.001) (H4 supported). Stakeholder behavior (*β* = 0.369, *p* < 0.001) and stakeholder attitudes (*β* = 0.392, *p* < 0.001) were both positively influenced by stakeholder trust in government, indicating that H5 and H6 are supported. However, the regression coefficient of the path from stakeholder trust in government to stakeholder risk perceptions was 0.046 (*p* > 0.05), suggesting that stakeholder trust in government did not affect stakeholder risk perceptions, thus rejecting H7.

Risk perception is the strongest predictor of human behavior. According to the SEM results, stakeholder risk perception could positively influence stakeholder behavior (*β* = 0.197, *p* < 0.001) (H8 supported), stakeholder attitudes (*β* = 0.155, *p* < 0.01) (H9 supported), and stakeholder priorities (*β* = 0.132, *p* < 0.05) (H11 supported), while stakeholder risk perception did not affect stakeholder intentions (*β* = 0.050, *p* > 0.05) (H10 rejected). Stakeholder behavior (*β* = 0.187, *p* < 0.001) and stakeholder attitudes (*β* = 0.143, *p* < 0.01) were positively impacted by stakeholder priorities; H12 and H13 are accepted. In contrast, stakeholder priorities did not affect stakeholder motivation (*β* = 0.062, *p* > 0.05).

**Figure 3:**
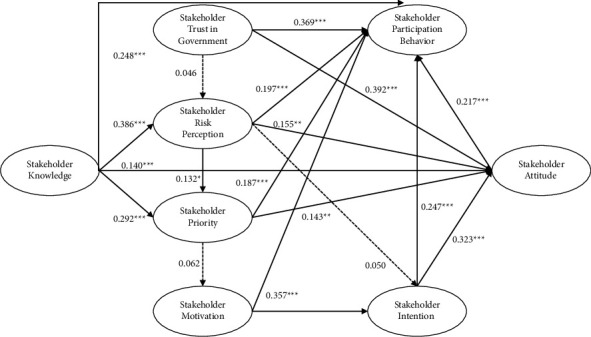
The results of the conceptual framework. *Note*. Only solid lines represent significant paths; ^*∗*^*p* < 0.05; ^*∗∗*^*p* < 0.01; ^*∗∗∗*^*p* < 0.001.

Hence, H14 is rejected. Stakeholder motivation could positively influence both stakeholder behavior (*β* = 0.357, *p* < 0.001) and stakeholder intentions (*β* = 0.442, *p* < 0.001), supporting H15 and H16, respectively. Furthermore, stakeholder behavior (*β* = 0.247, *p* < 0.001) and stakeholder attitudes (*β* = 0.323, *p* < 0.001) were positively impacted by stakeholder intentions; hence, H17 and H18 are accepted. Finally, the standardized path coefficient from stakeholder attitudes to stakeholder behavior was 0.217 (*p* < 0.001), suggesting that stakeholder attitudes significantly affected stakeholder behavior, thereby supporting H19.  Figure 3 presents the standardized regression weights of the causal paths in the model.

## 5. Discussion

One critical task of river restoration research is understanding why stakeholders are committed to participating in river restoration projects. Che et al. [102] noted that the success of river restoration depended on whether stakeholders were in favor of or opposed to restoration decisions. In this context, Furness [103] examined the relationship between participation in restoration projects and the natural environment, and Ceccon et al. [104] evaluated social involvement in restoration projects. Luyet et al. [105] presented a comprehensive framework for implementing stakeholder participation in environmental projects. Phalen [106] provided an account of people's reactions to restoration projects through theory about human behavior, motivation, and cognition. Few studies have been conducted to investigate the relationship between the drivers of stakeholder participation in river restoration projects. This study investigated the relationship between stakeholder attitudes, priorities and risk perceptions, trust in government, motivations, intentions, and knowledge and confirmed that all variables had a positive impact on stakeholder participatory behaviors. Such insights not only help to promote stakeholder participation in decision-making and shape attitudinal interventions but also have the potential to contribute to the success of river restoration projects.

The results reveal the positive impact of stakeholder attitudes on stakeholder participation behavior and stakeholder priorities. Attitude is a critical factor that influences participation behavior. The research findings suggest that individuals with positive attitudes toward the MSRP are more likely to be willing to participate and that attitudes toward river restoration projects are an underlying driver of stakeholder prioritization. This study broadens the existing discourse to facilitate stakeholder participation in river restoration decision-making, even though engaging laypeople in risk management is not an easy task. By clarifying the interaction between stakeholder trust in government decisions, risk perceptions, and behaviors with stakeholder priorities and participation behaviors, the importance of consistent participation processes with stakeholder priorities was emphasized.

The hypothesis that stakeholder risk perceptions positively influence stakeholder participation behavior was confirmed. Risk perception can influence stakeholder participation behavior, and the notion that, if stakeholders have an awareness of frequent flooding problems, they are more likely to participate in river restoration projects was reiterated, which is consistent with the findings of Hoti et al. [107] and Van Heel et al. [19]. Huang et al. [108] recommended using nature-based solutions or adaptive management approaches to reduce flood risk while mitigating the effects of climate change.

This study confirmed that knowledge can influence people's attitudes and behaviors. As shown in Table 1, less than half of the stakeholders have knowledge about participation in decision-making. Decision-makers cannot assume that all stakeholders have an understanding of how to participate in decision-making [109]. Hence, it is imperative to adopt the “social learning” approach to educate and train stakeholders to help them identify existing problems and develop the necessary knowledge to increase their understanding and preparedness for a project. Information access is also a prerequisite for improving people's ability to participate in decision-making [49]. Access and timely information are indispensable for motivating stakeholders to participate.

The study further revealed potential reasons that led to the rejection of the research hypotheses. The theoretical framework of “Protection Motivation Theory (PMT)” proposed by Rogers [110] provides compelling insights for rejecting H7, i.e., stakeholder trust in government decisions did not have any positive impact on stakeholder risk perceptions. Even if stakeholders trust government decisions on an emotional level, this does not change their perceived risk levels. Furthermore, the concept of risk perception is a multifaceted cognitive process covering both cognitive and affective dimensions, not just trust in a specific institution. Producing these results could be since trust in government decisions is inherently highly subjective and most decisions really do not focus on people's real concerns. H10 posits that stakeholder risk perception significantly affects stakeholder intentions. However, a path coefficient of *β* = 0.050 and a *p* value >0.05 exceeded the customary significance threshold and repudiated H10. According to Ajzen [56] TPB, intentions are shaped by a constellation of cognitive factors, including attitudes, subjective norms, and perceived behavioral control. While the risk perception may be a salient factor, it is not the sole determinant of intentions.

The path coefficient, *β* = 0.062 (*p* > 0.05), substantiates the rejection of H14 and establishes that stakeholder priorities do not exert a statistically significant influence on stakeholder motivation. The rejection of H14 was consistent with Ryan and Deci's [111] self-determination theory (SDT). SDT assumes that motivation is not a monolithic construct influenced by various psychological factors, including autonomy, competence, and relatedness. SDT emphasizes the role of autonomous motivation and states that stakeholders participate in river restoration projects because they find them inherently rewarding and in alignment with their values. Stakeholder priorities may be substantially affected when the priorities are consistent with their intrinsic values. On the contrary, if stakeholder priorities are perceived as incongruent with their value, their motivational impact may be limited. These outcomes collectively highlight the importance of considering the multifaceted nature of stakeholder behavior, motivations, and perceptions within the context of river restoration to help understand the relationship between the drivers that facilitate stakeholder participation behavior.

## 6. Conclusions

This research utilizes the MSRP in TC as an empirical lens to explore the relationship between the drivers behind stakeholder participation in river restoration projects, thus contributing to the ongoing discussion about how to incentivize stakeholder participation.

On theoretical grounds, this research confirms the applicability of the TPB in the field of stakeholder participation and reinforces the explanatory power of the TPB model by incorporating additional variables such as stakeholder knowledge, trust in government, risk perception, and priority. The research further validates the centrality of attitudes, knowledge, risk perception, and motivation as drivers of stakeholder behavior and their priorities. Notably, the results of this research are somewhat inconsistent with those of previous academics, e.g., stakeholder trust in the government has a minimal impact on their perceived risk, but the impact of stakeholder motivation on their risk perception and intention is magnified, thus revealing the complexity of stakeholder attitudes and behaviors in different contexts.

In a pragmatic sense, the insights presented in this study allow practitioners (local governments, decision-makers, and project managers) to gain a deep understanding of stakeholder participation behaviors since these insights not only helped restore the Moat System to an optimal state but also helped cultivate a sense of responsibility and ownership from stakeholders. The research findings illuminated effective avenues to promote stakeholder participation in decision-making for river restoration projects, thereby contributing to the successful outcome of river restoration projects.

Although this study has made progress in motivating stakeholders' participation behavior, there are still some limitations. First, this study only adopted a quantitative survey approach and did not consider a more comprehensive perspective. Bollen and Stine [112] state that SEM remains largely confirmatory rather than exploratory. The confirmatory nature of SEM may not capture emerging constructs. Furthermore, it must be recognized that the current research may have gaps in the impact of sociodemographic variables, lacking preliminary exploration of sociodemographic factors and critical research variables. Therefore, to expand the applicability of the current research findings, this study follows the recommendation of Bollen and Pearl [113] that, in the next stage, the research will strive to combine qualitative interviews or focus groups with quantitative surveys to supplement the quantitative research data. Meanwhile, the next step of this research will be devoted to exploring the impact of sociodemographic variables on other research variables throughout the study. The statistical control for the effects of the variables will also be taken into account.

## Figures and Tables

**Table 1:** Participant characteristics.

Attributes	Description	Frequency	Percentage (%)
Gender	Male	240	50.7
	Female	233	49.3

Age	18–25	105	22.2
	26–35	173	36.6
	36–45	81	17.1
	46–55	86	18.2
	55+	28	5.9

Education level	Primary school	95	20.1
	Secondary school	95	20.1
	Bachelor's degree	178	37.6
	Master's degree	85	18.0
	PhD+	20	4.2

Employment status	Civil servant	188	39.7
	Self-employed	85	18.0
	Scholar	8	1.7
	Worker	87	18.4
	Others	105	22.2

Years	Less than 1 year	94	19.9
	2–7 years	100	21.1
	8–13 years	88	18.6
	14–19 years	87	18.4
	More than 20 years	104	22.0

Familiarity with participation in decision-making	Not at all familiar	83	17.5
	Slightly familiar	105	22.2
	Moderately familiar	92	19.5
	Very familiar	96	20.3
	Extremely familiar	97	20.5

Flood management as a restoration priority	Approve	86	18.2
	Strongly approve	113	23.9
	Neutral	78	16.5
	Disapprove	91	19.2
	Strongly disapprove	105	22.2

**Table 2:** Measurement model results.

Construct	Item	Loading	CR	AVE	Cronbach's *α*
Stakeholder knowledge (SK)	(1). I have received full information regarding participation in decision-making	0.787	0.901	0.647	0.905
	(2). I have enough knowledge to participate in decision-making	0.793			
	(3). I learned about the process of participating in decision-making	0.806			
	(4). I was aware of my role in the decision-making process	0.802			
	(5). I know the significance of my participation in decision-making	0.832			

Stakeholder trust in government (ST)	(1). I think the decision of the government is trustworthy	0.807	0.902	0.605	0.901
	(2). I have confidence in the competence of decision-makers	0.775			
	(3). I am satisfied with the current decision-making process	0.764			
	(4). I believe the decision-making process is fair in Tianchang city	0.801			
	(5). I think the decision-making process is open and transparent in Tianchang city	0.758			
	(6). I think the decision-making in Tianchang is cross-disciplinary	0.759			

Stakeholder risk perception (SRP)	(1). I think the flooding problem of the Moat System is very serious	0.826	0.841	0.638	0.823
	(2). I think the flooding issue with the Moat System is threatening my life	0.816			
	(3). I agree with flood management as a priority for Moat System restoration projects	0.753			

Stakeholder priority (SP)	(1). Flooding management	0.823	0.901	0.647	0.902
	(2). Strengthen education and learning	0.819			
	(3). Strengthen legislation	0.803			
	(4). Ensure health and well-being	0.777			
	(5). Improve the quality of decision-making	0.798			

Stakeholder motivation (SM)	(1). I have the right to be part of the decision-making	0.812	0.898	0.637	0.884
	(2). I had a duty to participate in the decision-making	0.802			
	(3). I feel full confidence when I participate in decision-making	0.780			
	(4). I believe that my participation can influence decisions	0.801			
	(5). I have the responsibility to participate in decision-making	0.797			

Stakeholder intention (SI)	(1). Give me the possibility to learn new skills	0.768	0.888	0.612	0.883
	(2). Allow me to share my thoughts with more people	0.792			
	(3). Let decision-makers know and consider my ideas	0.777			
	(4). Obtain relevant knowledge and experience to participate in decision-making	0.774			
	(5). Gaining a sense of ownership	0.800			

Stakeholder attitude (SA)	(1). I am interested in Moat System restoration projects	0.780	0.892	0.623	0.811
	(2). I am concerned with Moat System restoration projects	0.798			
	(3). I know all about Moat System restoration projects	0.767			
	(4). I think the Moat System restoration project is beneficial to my life	0.802			
	(5). I am aware of the potential impact of Moat System restoration projects	0.797			

Stakeholder behavior (SB)	(1). I have experience participating in decision-making	0.781	0.889	0.615	0.870
	(2). I am willing to contribute my resources to participate in decision-making	0.781			
	(3). I am trained to participate in decision-making	0.764			
	(4). I can work with people in different roles	0.803			
	(5). I can encourage others to participate in decision-making	0.791			

**Table 3:** Discriminant validity.

Construct	SK	ST	SRP	SP	SM	SI	SA	SB
SK	**0.804**							
ST	0.056	**0.778**						
SRP	0.340	0.059	**0.799**					
SP	0.310	0.124	0.215	**0.804**				
SM	0.107	0.119	0.123	0.047	**0.798**			
SI	0.128	0.120	0.080	0.064	0.402	**0.782**		
SA	0.278	0.414	0.254	0.263	0.140	0.362	**0.789**	
SB	0.466	0.506	0.407	0.394	0.512	0.505	0.614	**0.784**

*Note*. The bold numbers indicate the square root of AVE.

**Table 4:** Goodness-of-fit of model.

	Chi-square	df	Chi-square/df	SRMR	RMSEA	TLI	CFI
Recommended value	—	—	<3	<0.10	<0.05	>0.9	>0.9
Measurement model	762.402	674	1.131	0.031	0.017	0.991	0.992
Structural model	791.242	682	1.160	0.054	0.018	0.989	0.990

**Table 5:** Results of hypothesis testing.

Path	Path direction	Nonstandard coefficient	SE	*Z* (CR value)	*p* value	Standardized coefficient	Result
H1	SK ⟶ SB	0.220	0.029	7.678	^*∗∗∗*^	0.248	Accepted
H2	SK ⟶ SRP	0.403	0.055	7.319	^*∗∗∗*^	0.386	Accepted
H3	SK ⟶ SA	0.132	0.047	2.806	0.005	0.140	Accepted
H4	SK ⟶ SP	0.308	0.058	5.297	^*∗∗∗*^	0.292	Accepted
H5	ST ⟶ SB	0.332	0.031	10.720	^*∗∗∗*^	0.369	Accepted
H6	ST ⟶ SA	0.376	0.045	8.389	^*∗∗∗*^	0.392	Accepted
H7	ST ⟶ SRP	0.048	0.052	0.919	0.358	0.046	Rejected
H8	SRP ⟶ SB	0.168	0.027	6.281	^*∗∗∗*^	0.197	Accepted
H9	SRP ⟶ SA	0.141	0.045	3.109	0.002	0.155	Accepted
H10	SRP ⟶ SI	0.045	0.044	1.026	0.305	0.050	Rejected
H11	SRP ⟶ SP	0.133	0.056	2.381	0.017	0.132	Accepted
H12	SP ⟶ SB	0.158	0.025	6.376	^*∗∗∗*^	0.187	Accepted
H13	SP ⟶ SA	0.129	0.042	3.070	0.002	0.143	Accepted
H14	SP ⟶ SM	0.060	0.050	1.209	0.227	0.062	Rejected
H15	SM ⟶ SB	0.309	0.029	10.583	^*∗∗∗*^	0.357	Accepted
H16	SM ⟶ SI	0.402	0.047	8.466	^*∗∗∗*^	0.442	Accepted
H17	SI ⟶ SB	0.235	0.033	7.209	^*∗∗∗*^	0.247	Accepted
H18	SI ⟶ SA	0.327	0.047	7.005	^*∗∗∗*^	0.323	Accepted
H19	SA ⟶ SB	0.203	0.034	5.925	^*∗∗∗*^	0.217	Accepted

*Note*: ^*∗∗∗*^*p* < 0.001.

## Data Availability

The data presented in this study are available on request from the corresponding author.
